# Evidence for virus-associated recapping behaviour in honey bees (*Apis mellifera*) with differential detection sensitivity between varroa-resistant and non-resistant colonies

**DOI:** 10.1038/s41598-026-44836-3

**Published:** 2026-03-26

**Authors:** Amélie Noël, Cathelijne G. A. Boer, Séverine D. Kotrschal, Joachim R. de Miranda, Naomi Keehnen, Barbara Locke

**Affiliations:** 1 Honey Bee Research Centre, Department of Ecology, SLU, Uppsala, Sweden; 2 Behavioural Ecology, Wageningen University and Research, Wageningen, The Netherlands; 3 Wageningen Plant Research, Wageningen University and Research, Wageningen, The Netherlands; 4 SciLifeLab, Department of Medical Sciences, Uppsala University, Uppsala, Sweden

**Keywords:** Deformed Wing Virus, Sacbrood Virus, Varroa-Sensitive Hygiene, hygienic behaviour, parasite-host interaction, Ecology, Ecology, Microbiology

## Abstract

Social immunity is vital for protecting honey bee colonies from pathogens and parasites. Among these threats, the parasitic mite *Varroa destructor* is particularly devastating, both by weakening parasitized bees and by transmitting several potentially lethal viruses, most notably *Deformed Wing Virus* (DWV). To counteract varroa and the damage caused by viral epidemics, honey bees exhibit complex, socially organized hygienic behaviours, including Varroa Sensitive Hygiene (VSH), in which workers detect and remove varroa infested brood. A related behaviour, known as recapping, involves workers opening, inspecting, and re-sealing brood cells. While the triggers for VSH are increasingly understood, the factors driving recapping remain largely unexplored, especially the potential role of brood viral infections. This study investigates the relationship between brood viral infections and recapping, and whether this relationship differs between naturally varroa-resistant and non-resistant colonies. Building on earlier work linking varroa-parasitized brood to recapping, our results provide evidence that viruses may also play a role in this behaviour. These findings suggest that worker bees may be able to detect changes induced by different virus infections and modulate recapping accordingly, highlighting a nuanced interplay between pathogen pressure and social immunity.

**Supplementary Information:**

The online version contains supplementary material available at 10.1038/s41598-026-44836-3.

## Introduction

Social immunity plays a crucial role in protecting group-living organisms, particularly eusocial insects, from pathogens and diseases^1^. This immunity can be either preventative, for example ingesting or using anti-microbial substances, or curative, for example expelling sick individuals from the colony. Either way, social immunity is always employed to protect the community as a whole from disease and pathogen transmission^2^. One of the most fascinating examples of curative social immunity can be found in the highly organized eusocial societies of honey bees (*Apis mellifera*). Honey bees exhibit complex social behaviours that contribute to the overall health and survival of their colonies^3,4^. One aspect of honey bee social immunity that has received significant interest is hygienic behaviour, of which there are several types. These behaviours enhance colony health and productivity either directly, by removing parasites, pests or disease agents, or indirectly, by expelling dead or diseased individual bees from the colony. Ultimately, the aim is to reduce the overall parasite-pathogen burden in the colony and limit spread of pests and pathogens within the nest.

Honey bees are hosts to numerous pathogens, pests and parasites. Among these, the most damaging is the parasitic mite *Varroa destructor*^5^. This mite has become a significant concern due to its devastating impact on honey bee colonies. The varroa mite feeds on the fat body and haemolymph of both adult honey bees and the capped brood, *i.e.* the immature developmental stages during metamorphosis from larva to adult bee^6^, weakening the bee’s individual immunity and making them more susceptible to other diseases and infections. During feeding, the mite acquires and transmits a range of common honey bee viruses which, when transmitted through this vector route, generate highly elevated titres in the affected bee, leading to delayed development, malformation and premature death of the individual bee^7^. Varroa mites are highly efficient vectors for transmitting two particularly devastating virus complexes: *Deformed Wing Virus* (DWV) and *Acute Bee Paralysis Virus* (ABPV)^8–10^. DWV is comprised of at least four major strains; DWV-A, -B, -C and -D^11^ whose distinct vector-host relationships influence the evolution of their relative and absolute distributions^12–14^. ABPV is a complex of at least three major strains (ABPV, *Kashmir Bee Virus*, and *Israeli Acute Paralysis Virus*) whose relative and absolute distributions have been negatively affected by the emergence of DWV as the main varroa-transmitted virus^15–17^. Other honey bee viruses such as *Black Queen Cell Virus* (BQCV), *Lake Sinai Virus* (LSV), and *Sacbrood Virus* (SBV), can in theory be mechanically transmitted by varroa, but this does not lead to lethal epidemics threatening colony survival. Their main bee health impact is as opportunistic secondary infections, further debilitating already weakened bees and colonies^5,16,18^.

To fight against the varroa mite, some honey bee colonies have a specific hygienic behaviour targeting varroa-infested brood cells known as Varroa Sensitive Hygiene (VSH)^19^. This VSH behaviour consists of three distinct steps: (1) the detection of parasitized brood cells by worker bees; (2) the opening of the brood cell for inspection by the adult bees; and finally (3) the cleaning out of the cell’s contents, removing the infested brood and disrupting the mite’s normal reproductive cycle and effectively mitigating the mite population growth within the colony^20^. A variant of this behaviour is known as recapping behaviour, where worker bees perform the first and second steps of VSH but then instead of cleaning out the cell, they recap it, leaving the developing bee intact^21–23^. Recapping behaviour can also play a role in disrupting varroa reproduction and therefore is potentially an anti-varroa defence strategy interfering with mite reproduction yet without sacrificing the developing host bees^24–26^. However, the link between reduced varroa reproduction and recapping is not always clear^27^. Recapping has the advantage of being less energy-consuming for the colony than VSH, by avoiding social apoptosis^28^. While it has been established that the identification of varroa-parasitized cells involves the detection of semiochemical cues^29–34^, there is limited understanding of the specific triggers for the recapping component of this behaviour. Nevertheless, some compounds have been identified as potential inducers of recapping^32^ and chemical cues in brood caused by DWV infection have also been shown to trigger removal^33^.

Some honey bee colonies exhibit higher rates of brood cell recapping than others^25,35^. The naturally varroa-resistant population on Gotland, Sweden, represents one population where elevated recapping rates have been observed and associated with their mite-resistance trait of reduced mite reproductive success^25,26^. However, the causal relationship and relative contribution of recapping to mite-resistance traits remains unclear. Recapping can be inconsistent, and is likely context dependent, rather than population specific^35^. Therefore, the mechanistic role of recapping in resistance, in relation to populations, and in various environments, remains an active area of research. The Gotland varroa-resistant honey bee population also display high resilience to virus infections^36^ with higher tolerance (*i.e.* reduced damage) to certain virus infections (*e.g.* DWV) and effective resistance (*i.e.* lower virus burden) to other viruses (*e.g.* BQCV, SBV, and LSV) relative to non-resistant colonies^37,38^. Higher colony-level virus tolerance and resistance are obviously beneficial traits for colony-level survival under uncontrolled varroa infestation^36^ and have not yet been explored for associations with behavioural social immunity.

This study aims to enhance our understanding of how pupal virus infections may influence adult bee recapping behaviour, which is a key aspect of social immunity. In addition, the potential for differences in this association will be explored between varroa-resistant and non-resistant colonies which could help explain natural varroa-resistance and survival. This will be achieved by comparing the presence and virus loads of five major honey bee viruses in recapped versus non-recapped pupae across both mite-resistant and mite-susceptible colonies to provide insight into the multifaceted interactions between viral pathogens and colony-level defence mechanisms.

## Materials and methods

### Experimental design

**Fig. 1:**
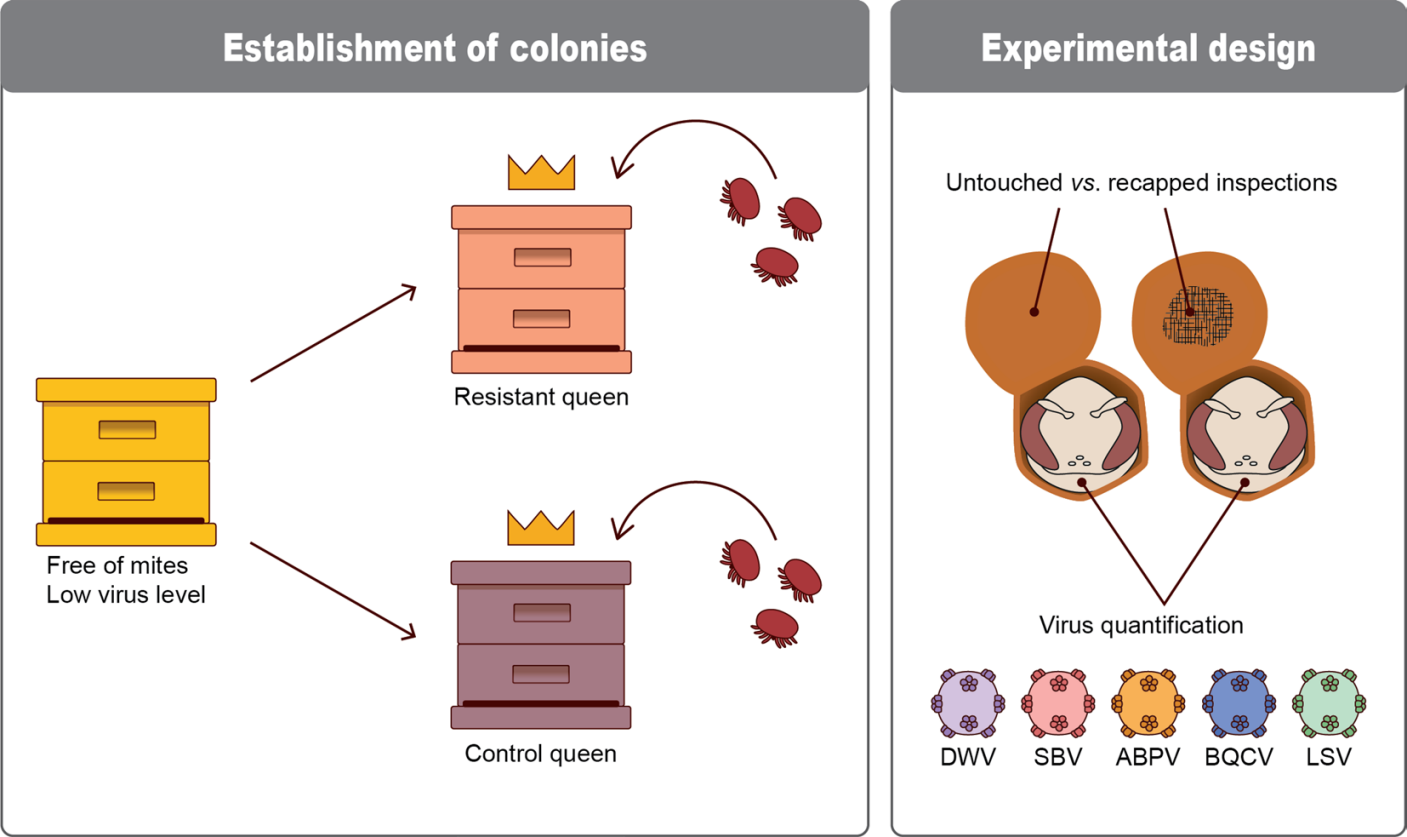
Illustration of the experimental design. DWV: Deformed Wing Virus, SBV: Sacbrood Virus, ABPV: Acute Bee Paralysis Virus, BQCV: Black Queen Cell Virus, LSV: Lake Sinai Virus.

This study was carried out during 2022 at the SLU Honey Bee Research Centre in Uppsala, Sweden. Five nearly broodless honey bee colonies were acquired from Åland, an island archipelago between Sweden and Finland which was still varroa-free when these experiments were conducted, with a very low incidence of bee viruses^13,39^. The colonies were split, with the queen-less split receiving a queen from the mite-resistant honey bee population on Gotland. This resulted in 10 test colonies whose adult bees were uniformly distributed, largely virus-free, and headed by queens from either Åland lineages, that had never been exposed to varroa and considered a non-resistant control group for experimental purposes, or from Gotland lineages, that have naturally survived varroa infestation for > 20 year and are essentially a mite-resistant population (Fig. 1). The colonies were allowed to settle for 6 weeks before the start of the experiments, to replace the adult bee population in the mite-resistant colonies with the progeny and (epi-)genetic characteristic of the introduced Gotland queen. Of these ten colonies, one of the non-resistant colonies was too weak to be included in the experiment and two of the resistant colonies suffered from queen acceptance failure. Therefore, four non-resistant and three resistant colonies were used in the experiment. The colonies were not treated against varroa or other diseases prior to the experiments.

To induce virus infections, test colonies were naturally infested with *Varroa destructor* by inserting a frame of varroa-infested drone brood from mite donor colonies (Fig. 1). Each test colony received two frames and infestation levels were monitored using the soapy water method on samples of approximately 300 adult bees^40^, in order to ensure that enough mites were available for invading pupal cells.

In total, 2095 cells were opened for inspection of recapping and varroa infestation. On average, recapped cells were about twice as likely to have a mite present than untouched cells, which agrees with previous research^26^. Since this study focused on the impact of viruses on the recapping behaviour, we selected 275 individual pupae, evenly distributed between untouched and recapped by adult bees, from either the control or resistant colonies for virus analysis (Fig. 1; Supplementary Table S1).

### Assessment of recapping behaviour

To study the recapping behaviour of each colony, an average of 40 brood cells containing pupae between eight- and eleven-days post-capping were gently opened using a scalpel and tweezers. A cell was characterised as recapped when a portion of the pupae’s silk cocoon was absent from the underside of the cell capping. The age of the pupae and the mite infestation status were noted. The sampled pupae were frozen at -20 °C for subsequent molecular analyses, as it does not impact the integrity of the samples^41^.

### Nucleic acid extraction

The pupae were homogenized in 800 µL TBS buffer + RNA250 using a bead-mill at 5,400 RPM for 90 s, repeated once (Precellys Evolution, Bertin Technologies). RNA was extracted from 100 µL homogenate using the Qiagen RNeasy^®^ Plant Mini Kit, eluted in 50 µL nuclease-free water, and either stored at -80 °C or immediately converted to cDNA. The cDNA was synthesized from 0.1 to 5 µg RNA using the First Strand cDNA Synthesis Kit (Thermo Fisher Scientific, #k1612) with random hexamers, diluted 10 times in AE buffer (10 mM TRIS.CL (9.0)/0.5 mM EDTA), and stored at -20 °C prior to RT-qPCR assays.

## RT-qPCR assays

RT-qPCR assays were performed following the protocol outlined in de Miranda *et al.* (2021)^42^ on a thermocycler (CFX Connect Real-Time System, Bio-Rad) using SsoFast EvaGreen Supermix (with a Sso7d dsDNA-binding protein) and primers specific for DWV, ABPV, LSV, BQCV, SBV, as well as for an exogenously added reference RNA (RNA250: 8 ng or 7,504,330,568 copies of RNA250, which is synthetic a 2,002-nt ssRNA with a molecular weight of 641,991 g/mol) of known absolute concentration used to normalize the virus data between samples^43^(Supplementary Table S2). Every assay was performed with 10 µL reaction containing 2 µL 1:10 diluted cDNA, 0.4 µL each primer (10 mM), 5 µL 2xEvaGreen Supermix, and 2.2 µL RNase-free water. Also included on each plate were duplicate columns each containing two negative Non-Template Controls (NTC) and a six step 10-fold dilution series of a synthetic plasmid of known absolute concentration (copies per µL) of the assay target, produced by Eurofins (Ebersberg, Germany) to enable absolute quantification. Thermocycling conditions were as follows: 95 °C for 3 min, followed by 40 cycles of 95 °C for 15 s and 58 °C for 20 s, followed by a Melting Curve analysis for amplicon verification, consisting of denaturation at 95 °C for 60 s, 55 °C for 60 s and 5 s incubations from 55 °C to 95 °C at 0.5 °C increments followed in each instance by a fluorescence reading.

Samples were classified as positive if the melting peak was consistent for the true amplicon and both duplicates displayed a cycle quantification (CQ) value between 0 and 35^16,44^. CQ values were determined at a fluorescence threshold of 200 RFU. For absolute quantification, standard curves were generated by plotting CQ values of the dilution series control against their log_10_-transformed template copy numbers per reaction. The resulting linear regression was used to estimate starting quantities (SQ values) for each target RNA in the cDNA reactions. The raw absolute viral RNA abundances (virus titres) per pupae were calculated by averaging the SQ values of the duplicate qPCR reactions. These values were convert to the amount of virus per individual bee, and simultaneously correct for individual differences between samples in the RNA extraction and cDNA synthesis steps, with the following equation:

$$\:{[virus/bee\:sample]\:=\:\mathrm{S}\mathrm{Q}}^{\mathrm{v}\mathrm{i}\mathrm{r}\mathrm{u}\mathrm{s}}/{\mathrm{S}\mathrm{Q}}^{\mathrm{R}\mathrm{N}\mathrm{A}250}\:\times\:[RNA250/bee\:sample]$$

Where:

[virus/bee sample] = the estimated amount of virus in the bee sample (in genome copies).

SQ^virus^ = the SQ value for the estimated amount of virus in 2 ul cDNA (in genome copies).

SQ^RNA250^ = the SQ value for the estimated amount of RNA250 in 2 ul cDNA (in genome copies).

[RNA250/Bee sample] = the amount of exogenous RNA250 added to the bee sample prior to homogenization (in genome copies).

### Statistical analysis

Due to the absence of SQ value for RNA250, five samples were excluded from the analysis. Furthermore, due to the high risk of varroa escape from recapped cells^24^, the varroa status of recapped cells in this study was considered unreliable. Varroa status was therefore excluded as a factor in the analysis of the virus data. To study the effect of the presence of each virus on the recapping behaviour, generalised linear mixed models were used with a binomial distribution. The response variable was the viral presence in each pupa, with colony type (resistant/control), cell type (untouched/recapped), and their interaction as fixed effects, and colony identity as a random effect. To study the effect of the amount of each virus on the recapping behaviour, the genome copy number of each virus in the infected pupae was log_10_-transformed and fitted on a linear mixed-effects model. Fixed effects included colony type, cap status, and their interaction, with colony identity as a random effect. For both presence and viral copy number analysis, the significance of fixed effects for each model was evaluated using ANOVA. Pairwise comparisons of estimated marginal means were performed with the contrast method to identify significant differences between specific groups (Table 1), applying a Šidák correction for multiple testing. The pairwise comparisons included: untouched vs. recapped cells within each type of colony (resistant or control), recapped cells between colony type, and intact cells between colony type. All statistical analyses were carried out on R v4.4.1., with the packages “ade4” (v1.7-22), “performance” (v0.13.0), “lme4” (v1.1-35.5), “car” (v 3.1-2), and “emmeans” (v1.10.3).

## Results

**Fig. 2:**
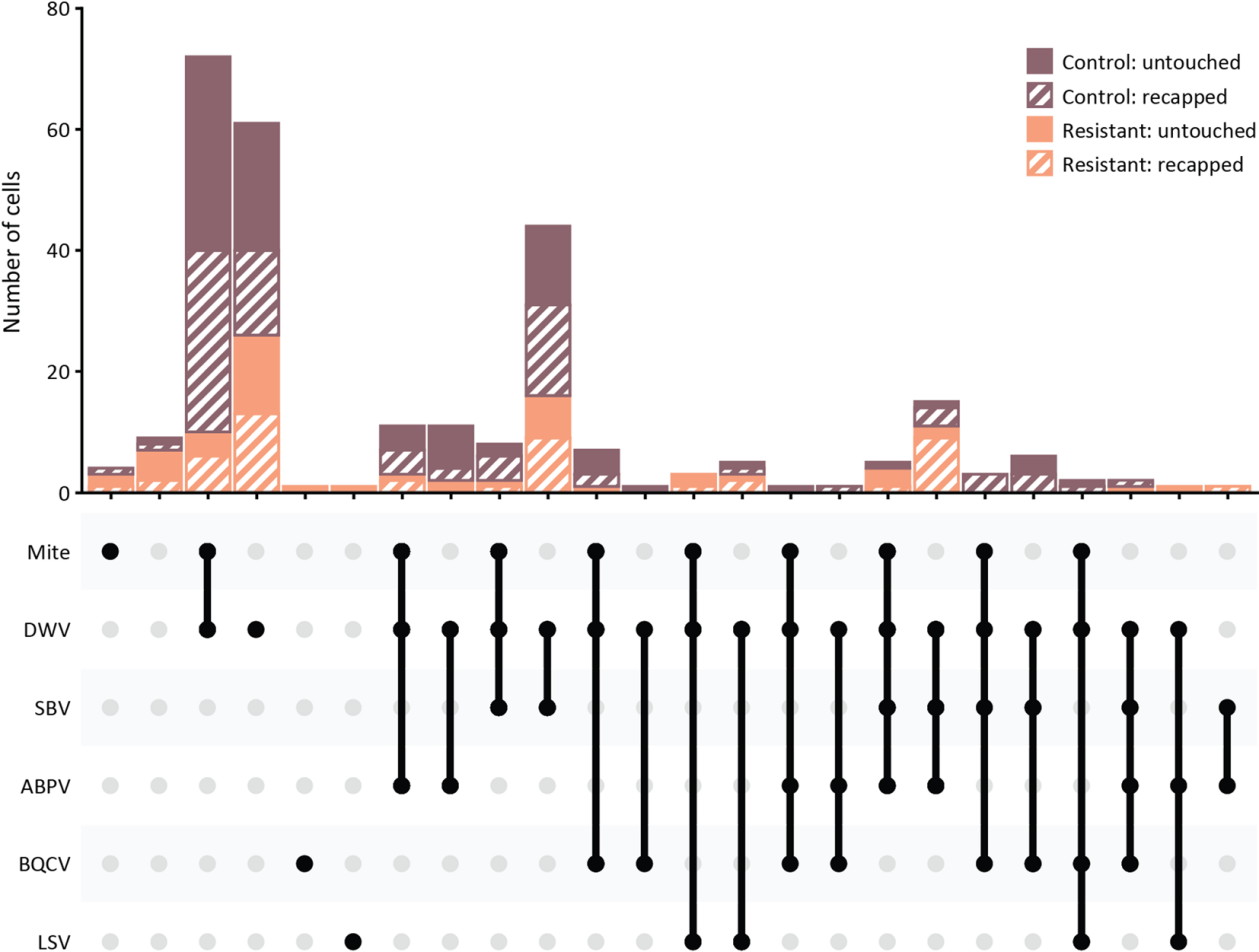
Mite infestation and viral (co-)presence in untouched and recapped pupae from control and resistant colonies. Black dots represent presence of the mite and/or the various viruses, and linked dots represents co-presence. Brown: control colonies, orange: resistant colonies, plain: untouched cells, stripes: recapped cells.

Virus presence in the pupae varied significantly among the five screened viruses. As expected, DWV was the most prevalent (94.2%), followed by SBV (30.5%) (Fig. 2). The remaining viruses were detected at lower rates (ABPV: 17.5%, BQCV: 8.73% and LSV: 4.36%) (Fig. 2). This result is representative of the viral landscape in Sweden and is consistent with other studies^12,45^.

**Fig. 3:**
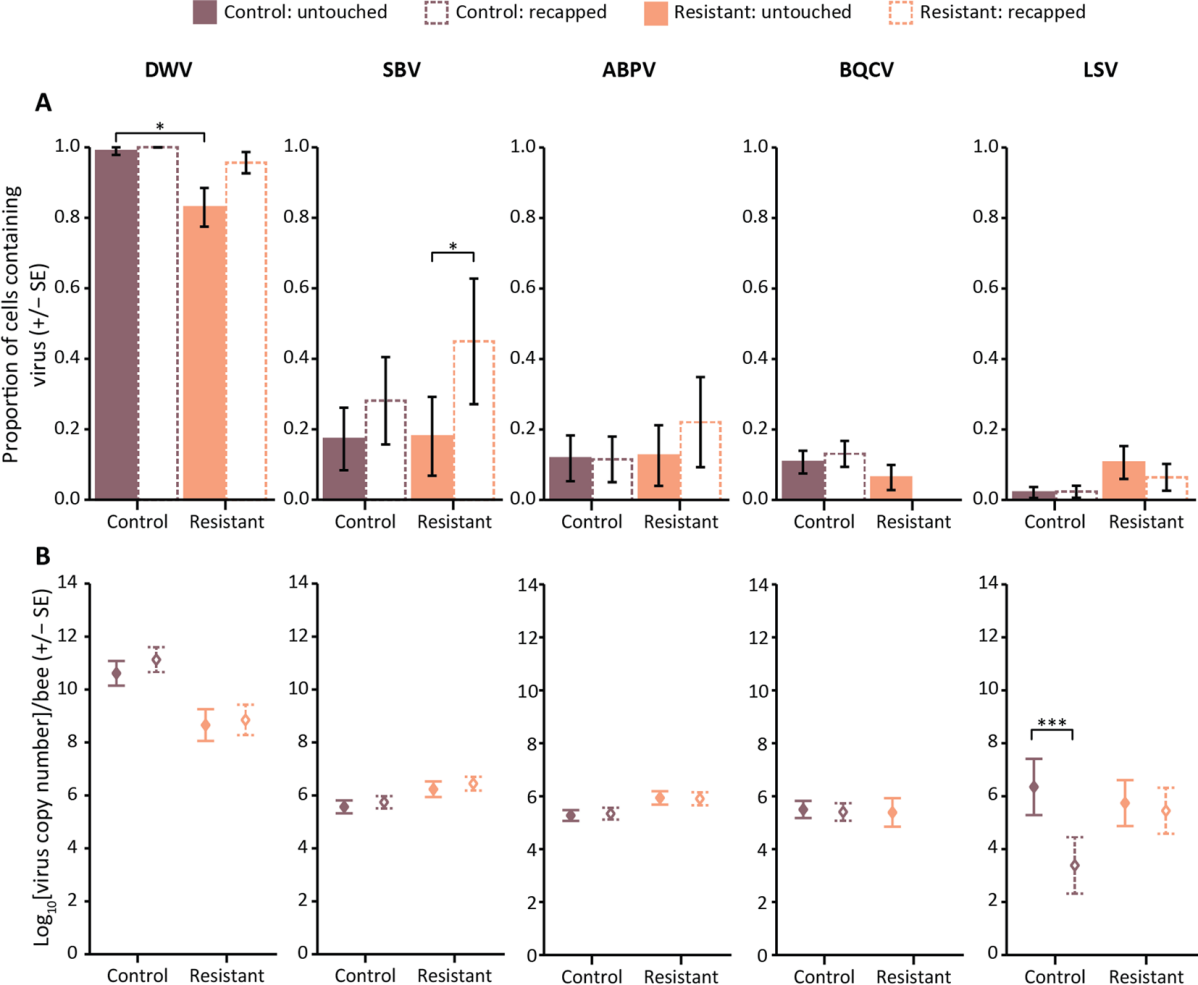
Virus infections in 7- to 11-day old post-capping untouched and recapped pupae. Estimated marginal means (+/- SE) of (**A**) the prevalence and (**B**) the amount in infected pupae of DWV (*Deformed Wing Virus*), SBV (*Sacbrood Virus*), ABPV (*Acute Bee Paralysis Virus*), BQCV (*Black Queen Cell Virus*), and LSV (*Lake Sinai Virus*). Brown: control colonies, orange: resistant colonies, plain: untouched cells, dashed: recapped cells. Statistics from pairwise comparisons; *: p-value ≤ 0.05, ***: p-value < 0.001.

When considering virus presence according to colony type and cell type, there was a significant difference for DWV between colony type (varroa-resistant or control; χ^2^ = 7.43, d.f. = 1, *p* = 0.006), but not for cell type (recapped or untouched; χ^2^ = 3.37, d.f. = 1, *p* = 0.066) or the interaction between both factors (χ^2^ = 0.00, d.f. = 1, *p* = 0.969). A statistically significant difference was observed in the proportion of untouched cells between resistant and control colonies (Table 1A, Fig. 3A). This was mostly because nearly all pupae in the control colonies, untouched or recapped, were infected with DWV while the resistant colonies had overall a much lower proportion of DWV infected individuals, with a considerably more frequent presence of DWV in recapped pupae than in untouched pupae. By contrast, there was a significant difference in SBV presence between cell type (χ^2^ = 7.83, d.f. = 1, *p* = 0.005), but not between colony type (χ^2^ = 0.24, d.f. = 1, *p* = 0.624) or the interaction between cell type and colony type (χ^2^ = 1.11, d.f. = 1, *p* = 0.291). Moreover, while in both varroa-resistant and non-resistant colonies, there was a greater proportion of SBV-infected pupae in recapped cells than in untouched cells, this difference was considerable larger and statistically significant in varroa-resistant colonies (Table 1A, Fig. 3A). ABPV, BQCV and LSV were all only detected at low prevalences (Figs. 2 and 3A). There was no significant difference in the presence of these three viruses between either colony type (χ^2^ = 0.25, d.f. = 1, *p* = 0.621), cell type (χ^2^ = 0.61, d.f. = 1, *p* = 0.435) or their interaction (χ^2^ = 1.04, d.f. = 1, *p* = 0.307). While in the resistant colonies ABPV was more often present in recapped than untouched cells, none of the effects were statistically significant (Table 1A, Fig. 3A). Similarly, none of the factors or the interaction of the factors were significant for the presence of BQCV (colony type: χ^2^ = 0.69, d.f. = 1, *p* = 0.405; cap status: χ^2^ = 0.23, d.f. = 1, *p* = 0.631; interaction: χ^2^ = 0.00, d.f. = 1, *p* = 1). Nevertheless, its prevalence was slightly higher in non-resistant colonies than resistant colonies with no real difference between untouched and recapped cells (Table 1A, Fig. 3A). None of the recapped cells in the resistant group were infected with BQCV. While LSV presence was significant between colony type (χ^2^ = 4.46, d.f. = 1, *p* = 0.035), it was not the case between cell types (χ^2^ = 0.25, d.f. = 1, *p* = 0.620) or the interaction between colony type and cell type (χ^2^ = 0.26, d.f. = 1, *p* = 0.607). LSV was slightly more prevalent in resistant colonies, particularly in untouched cells in comparison with recapped cells, without being significant (Table 1A, Fig. 3A).

**Table 1:** P-values of pairwise comparisons of estimated marginal means for (**A**) virus prevalence between different categories of pupae and (**B**) virus titres between different categories of infected pupae. P-value ≤ 0.05 are represented in bold.

A. Virus prevalence					
Comparisons	DWV	SBV	ABPV	BQCV	LSV
Untouched cells: control group vs. resistant group	**0.025**	1.000	1.000	0.875	0.207
Recapped cells: control group vs. resistant group	1.000	0.898	0.887	1.000	0.708
Control group: untouched cells vs. recapped cells	1.000	0.305	1.000	0.981	1.000
Resistant group: untouched cells vs. recapped cells	0.240	**0.055**	0.588	1.000	0.926

B. Virus titres of infected pupae					
Comparisons	DWV	SBV	ABPV	BQCV	LSV
Untouched cells: control group vs. resistant group	0.128	0.407	0.336	0.981	0.990
Recapped cells: control group vs. resistant group	0.068	0.335	0.484	-	0.647
Control group: untouched cells vs. recapped cells	0.442	0.786	0.995	0.968	**< 0.001**
Resistant group: untouched cells vs. recapped cells	0.992	0.808	0.999	-	0.147

For virus titres (Fig. 3B), there was a significant difference in colony type for DWV (χ^2^ = 9.646, d.f. = 1, *p* = 0.002), SBV (χ^2^ = 4.406, d.f. = 1, *p* = 0.036) and ABPV (χ^2^ = 4.53, d.f. = 1, *p* = 0.033) although with no significant difference between cell type (DWV: χ^2^ = 2.12, d.f. = 1, *p* = 0.146; SBV: χ^2^ = 1.94, d.f. = 1, *p* = 0.164; ABPV: χ^2^ = 0.02, d.f. = 1, *p* = 0.894) and thus also no interaction between colony type and cell type (DWV: χ^2^ = 0.27, d.f. = 1, *p* = 0.602; SBV: χ^2^ = 0.02, d.f. = 1, *p* = 0.899; ABPV: χ^2^ = 0.13, d.f. = 1, *p* = 0.723). DWV titre was marginally higher in recapped cells from the control colony group than resistant colony, approaching statistical significance (Table 1B, Fig. 3B). While there were slightly higher amounts of SBV and ABPV in resistant colonies compared to the non-resistant colonies, this was not significant in the pairwise comparisons (Table 1B, Fig. 3B). There is no difference in amount of BQCV between either colony type or cell type (colony type: χ^2^ = 0.03, d.f. = 1, *p* = 0.856; cap status: χ^2^ = 0.05, d.f. = 1, *p* = 0.807), and no interaction was observed, as there were no BQCV-infected recapped cells in the resistant population (Table 1B, Fig. 3B). Both cell type and its interaction with colony type were significant for the amount of LSV virus in LSV-infected cells (colony type: χ^2^ = 0.043, d.f. = 1, *p* = 0.836; cell type: χ^2^ = 163.24, d.f. = 1, *p* < 0.001; interaction: χ^2^ = 200.09, d.f. = 1, *p* < 0.001) (Fig. 3B). This is most likely due to the much lower levels of LSV in recapped pupae from non-resistant colonies, relative to the untouched pupae (Table 1B, Fig. 3B).

## Discussion

The ability of honey bee colonies to combat diseases and parasites is fundamental to their survival. In addition to individual immune responses, honey bees uniquely rely on social immunity behaviours, yet the mechanisms by which these behaviours contribute to bee health remains not totally understood. In this study, we examined the relationship between viral infections in pupae and adult bee recapping behaviour. Several studies have demonstrated an association between varroa-parasitized brood cells and recapping behaviour^26,35,46,47^. The results of our study extend this knowledge by showing that recapping may also occur in the context of viral infections and that the presence, titres, and species of virus may influence recapping behaviour. Virus effects on recapping were more pronounced in varroa-resistant colonies than in non-resistant colonies, specifically in SBV prevalence, with similar but non-significant trends for DWV and ABPV. There was significantly higher LSV titres in untouched pupae compared to recapped pupae in the control colonies, but it is not possible to make inferences on the relationship between LSV and recapping because this virus was found at very low prevalence throughout the experiment. BQCV did not show an effect on recapping, but it is interesting to note that none of the recapped cells from the resistant population carried BQCV, whereas all other pupae groups carried it. Previous studies have shown that BQCV infection decreases towards the end of the season in colonies of the Gotland population, while it simultaneously increases in control colonies^36,48^. The absence of pupae with BQCV in recapped cells from the resistant colonies in this study may suggest systematic cleaning of the BQCV-infected pupae by their workers but requires further investigation.

The amount of virus generally had little effect on recapping compared to the mere presence, or prevalence, of a virus. The largest difference in viral titre was observed for DWV, which occurred at substantially lower amounts in pupae from varroa-resistant colonies than in pupae from non-resistant colonies overall. Conversely, no statistically significant intra-population effect of the presence of DWV was observed on recapping behaviour, despite a small trend of a higher DWV prevalence in recapped *vs.* untouched pupae in the mite-resistant population. In fact, the lack of a significant difference in DWV prevalence and virus-load between untouched and recapped cells observed here is consistent with previous studies reporting no significant variation in DWV levels between VSH-targeted and non-targeted brood cells^49^. Given the global ubiquity of DWV in honey bee colonies and its strong association with varroa mite infestation, the lack of a detectable effect on recapping behaviour by DWV is perhaps not surprising^5,9,10,14^. However, pupae infected with the more virulent DWV-B strain are more likely to be detected and removed by worker bees than those infected with DWV-A^50,51^. In Sweden, DWV-A is the predominant circulating strain found in honey bee populations^12,38,52,53^. This dominance of the less virulent DWV-A lineage may therefore reduce the selective pressure for targeted removal of DVW-infected brood, potentially explaining the lack of difference in DWV load between recapped and untouched brood cells in this study.

Enhanced detection and removal of infected brood would impose a strong selective pressure against high virulence of DWV in the Gotland population. Under a standard virulence-transmission trade-off, highly virulent strains incur fitness costs because they induce host immune responses that ultimately reduce transmission opportunities, while less virulent pathogens, causing milder infections, are less likely to be detected and can persist and transmit for longer^54^. Consequently, selection in a host population with sensitive detection is expected to favour reduced virulence. DWV’s epidemiological success is largely facilitated by its already relatively low host virulence at the pupal stage. Low virulence allows the host pupa to survive until adulthood, increasing transmission opportunities for DWV, whereas highly virulent virus strains killing the host are removed from the colony by adult worker defence behaviours and ultimately reduce the pathogen transmission optimum. Therefore, a heightened sensitivity to DWV in the mite-resistant population could contribute to the natural long-term survival of this population either through increased host resistance (higher detection sensitivity) or reduced pathogen virulence (because of host selection pressures), but this remains to be studied. A comparable phenomenon was recently described in the free living, varroa-surviving honeybee colonies of the Arnot Forest in upstate New York, where experimental infections demonstrated that DWV strains derived from these naturally varroa-surviving colonies were less virulent than those from surrounding managed colonies^55^.

This study also demonstrates a higher prevalence of SBV in recapped brood cells compared to untouched cells within the mite-resistant bee population on Gotland. This pattern may indicate that workers from resistant colonies are more likely to inspect, and recap, cells containing SBV-infected pupae. In contrast, workers from non-resistant colonies appear to exhibit less frequent inspection and recapping behaviour associated with SBV-infected pupae. SBV primarily infects and kills young brood (larvae and pre-pupae stages), often leading to the removal of highly infected individuals even before the capping by workers^10,15,56^. The Gotland mite-resistant population maintains comparatively low SBV levels, even decreasing SBV levels over the season^36,48,53^. Because SBV infection is associated with pollen-aversion behaviours that reduce foraging and resource intake^56,57^, limiting SBV titres in the colony would support improved colony performance and survival. The capacity to identify SBV-infected brood and assess infection levels may therefore represent a critical component of the SBV-resistance observed in this population^36^. Recapping could potentially represent a strategic compromise. Rather than sacrificing all infected brood, adult workers may preserve individual pupae with moderate infection levels that retain a chance for survival and the potential to develop into functional workers, even if they carry a slightly higher virus load than healthy individuals. This selective tolerance aligns with theoretical frameworks describing trade-offs between the energetic costs of social apoptosis, sacrificing an individual for the good of the colony, and the potential colony-level benefits of preserving partially infected, yet viable, individual brood^58,59^. This nuanced balance between pathogen control through social immunity and resource allocation underscores the complex evolutionary dynamics shaping social immunity in honey bees.

VSH relies on pupal-derived specific signals, particularly chemical signals emitted by infected or parasitized pupae, and eliciting a targeted response from worker bees. It has been suggested that recapping behaviour is similarly mediated by such signals. One proposed hypothesis is the existence of a “threshold” signal, that guides workers in deciding whether or not to recap the cell or remove the brood^32^, and is supported by evidence that cells are more likely to be recapped when the brood is less damaged^28^. In addition, several highly volatile compounds associated with recapping behaviour vary in concentrations in either varroa-parasitized or non-parasitized brood, helping worker bees to assess and differentiate brood health status^32^. Chemical cues from DWV-infected pupae are also known to trigger VSH behaviour^33^. Recent studies further demonstrate that viral infection alters chemical signalling in honey bees^60,61^. For example, DWV and IAPV modify the pupal volatolome^60^, while DWV and BQCV reduce the emission of methyl oleate, a component of the queen retinue pheromone as well as of the brood ester pheromone^61^. These previous studies support the possibility that an olfactory threshold could exist in the context of viral infections, particularly for SBV in light of our findings. Worker bees may be capable of detecting a viral infection, opening the cell, and then assessing infection severity and determining the likelihood of whether a pupa is sufficiently healthy to develop into a viable adult. Such a threshold-based mechanism could allow workers to choose between removing or recapping brood, thereby optimizing colony health and resource allocation.

It is important to acknowledge a key limitation in any study investigating recapping behaviour: the total absence of comparative data on pupae from cells that were cleaned out by worker bees. Because viral load could not be measured in pupae that were cleaned out by hygienic behaviour, we were unable to determine whether a specific virus or virus titre increased the likelihood of either the recapping or removal behaviour after uncapping. This constraint highlights a broader methodological challenge in studying recapping behaviour in general, namely, the difficulty of preventing or controlling worker bee cleaning activity, which complicates efforts to isolate mechanisms associated solely with recapping.

In conclusion, this study suggests that viral infections may contribute to the variation observed in recapping behaviour. The elevated prevalence of SBV in recapped brood cells, relative to untouched cells, suggests that worker bees can detect the changes in pupal physiology and volatiles composition induced by SBV infection, and act accordingly. The other major proposed hypothesis is that bees from the Gotland mite-resistant population may be much more reactive to pupal changes induced by DWV infection than bees from the control colonies, resulting in recapping (and presumably also removal) behavioural responses to much lower DWV titres, reducing both DWV burden and associated varroa-related damage relative to control colonies. These findings highlight the complex interplay between viral infections and recapping behaviour, a key component of social immunity, and emphasize the need for further research to elucidate the mechanisms underlying this behaviour. Understanding these processes and interactions could inform the development of more effective and sustainable strategies for maintaining honey bee colony health.

## Supplementary Information

Below is the link to the electronic supplementary material.

Supplementary Material 1

## Data Availability

The datasets generated during the current study are available from the corresponding author on reasonable request.
